# Supplementary material for: Corrosion-influencing microorganisms in petroliferous regions on a global scale: systematic review, analysis, and scientific synthesis of 16S amplicon metagenomic studies
Source: PeerJ. 2023 Jan 13;11:e14642. doi: 10.7717/peerj.14642 (PMC9841911; doi:10.7717/peerj.14642)
Supplement: Supplemental Information 2 — This document explains 1. who are the two authors who performed the Search Strategy and identify them in our manuscript; 2. how disagreements were resolved, and identify the referee in our manuscript, 3. The rationale for conducting the systematic review / meta-analysis; 4. The contribution that it makes to knowledge in light of previously published related reports, including other meta-analyses and systematic reviews. [file peerj-11-14642-s002.docx]

**Systematic Review/Meta-Analysis Search**

**Please clarify who are the two authors who performed the Search Strategy and identify them in your manuscript:**

Database searches was carried out automatically, using scripts developed in our own research group by Glen Jasper Yupanqui Garcia (G.J.Y.G.). The selection of studies was performed by Joyce Dutra (J.D.) and Rosimeire Gomes (R.G.) and the divergences were solved by Mariana Santos Cardoso (M.S.C.).

**You should also describe how disagreements were resolved, and identify the referee in your manuscript:**

The divergences were solved as follows: After obtaining the documents (Joyce Dutra and Rosimeire Gomes), selected and non-selected files were merged, and the discrepancies were identified. Thus, these divergent documents were separated in one spreadsheet and forwarded to the third reviewer (Mariana Santos Cardoso), who judged whether the document would enter or not. In case of doubt, there was a census in the group of authors to decide whether the document should be remained in the final *corpus*, or removed.

**Systematic Review and/or Meta-Analysis Rationale**

**For systematic reviews / meta-analyses, authors need to provide the following information** :

**The rationale for conducting the systematic review / meta-analysis;**

The global annual costs of the corrosion of pipelines and other industrial equipment for oil and gas production and transport have been estimated at 2.5 trillion US dollars per year, according to the NACE International Study (2016), and it is estimated that between 10 and 20 % of corrosion occurrences involve MIC (Machuca & Salgar-Chaparro, 2019). MIC refers to corrosion that is affected by the presence and activity of microorganisms, which involves the establishment of biofilms that initiate and/or accelerate corrosive processes of electrochemical nature (Eckert, 2014; Videla, 1996; Zarasvand & Rai, 2014). MIC is difficult to predict and monitor due to the heterogeneity of biofilms distribution within industrial systems and because it induces localized corrosion, whose monitoring is much more challenging than general corrosion (Little & Lee, 2007; Roche, 2007). Operating expenditures for mitigating MIC include mechanical cleaning, pigging, injection of chemical products (e.g., biocides, biodispersants, corrosion inhibitors, O_2_ and H_2_S scavengers), line inspection, and microbiological and physicochemical analyses (Skovhus et al., 2017).

Microbiological analyses allow the evaluation of the microbiota present in a given environment, including those that are capable of causing or accelerating corrosion. Although cultivation-dependent methods are still the most widely used in the oil and gas industry, they are limited because cultivable lineages usually do not exceed 1.0 % of all microbial lineages present in environmental samples (Pedrós-Alió, 2011). Amplicon metagenomics (or metabarcoding) has been progressively used to substitute culture-dependent methods, even with the limitation of not providing direct information about the functional and metabolic capabilities of the evaluated communities (Douglas et al., 2020). The scientific literature on amplicon metagenomic in oil production and transport structures worldwide is highly scattered and the studies are usually extremely localized. Thus, the objective of the current systematic review was to evaluate the taxonomic composition and relative abundance of bacteria and archaea associated with the microbiologically influenced corrosion (MIC), and the prediction of their metabolic functions in different sample types from oil production and transport structures worldwide.

(**Note:** Cited References appear in the Reference section of the submitted manuscript).

**The contribution that it makes to knowledge in light of previously published related reports, including other meta-analyses and systematic reviews; eg**

Our study evaluated the microbial profile of samples associated with the oil industry (PW, IW, OW, OIL, and SD) in seven petroliferous regions, comprehending several countries all over the world. Many of these countries figure in the ranking of the top 15 oil producers in the world (IBP, 2020) and each petroliferous region has at least one country classified in this ranking (Table 2). This demonstrates, in general, that the current systematic review constitutes a representative sample of the world scenario, referring to the composition and abundance of microorganisms present in petroliferous regions.

The results showed that the sulfidogenic, methanogenic, acid-producing, and nitrate-reducing functional groups were the most expressive, all of which can be directly involved in MIC processes. There were significant positive correlations between microorganisms in the injection water (IW), produced water (PW), and solid deposits (SD) samples, and negative correlations in the PW and SD samples. Only the PW and SD samples displayed genera common to all petroliferous regions, *Desulfotomaculum* and *Thermovirga* (PW), and *Marinobacter* (SD). There was an inferred high microbial activity in the oil fields, with the highest abundances of (i) Cofactor, (ii) Carrier, and (iii) Vitamin Biosynthesis, associated with survival metabolism. Additionally, there was the presence of secondary metabolic pathways and defense mechanisms in extreme conditions. Competitive or inhibitory relationships and metabolic patterns were influenced by the physicochemical characteristics of the environments (mainly sulfate concentration) and by human interference (application of biocides and nutrients). Our worldwide baseline study of microbial communities associated with environments of the oil and gas industry will greatly facilitate the establishment of standardized approaches to control MIC.

(**Note:** Cited References appear in the Reference section of the submitted manuscript).
