# Supplementary material for: Corrosion-influencing microorganisms in petroliferous regions on a global scale: systematic review, analysis, and scientific synthesis of 16S amplicon metagenomic studies
Source: PeerJ. 2023 Jan 13;11:e14642. doi: 10.7717/peerj.14642 (PMC9841911; doi:10.7717/peerj.14642)
Supplement: Supplemental Information 3 [file peerj-11-14642-s003.docx]

| **Database** | **Keywords** |
| --- | --- |
| Web of Science | ALL=(((petroleum) OR (crude oil) OR (oilfield) OR (oil)) AND ((reservoir OR pipeline)) AND ((biocorrosion) OR (microbiologically influenced corrosion) OR (microbial corrosion) OR (MIC AND corrosion)) AND ((metagenomics) OR (16s rRNA))) |
| Scopus | ("petroleum" OR "crude oil" OR "oilfield" OR "oil") AND ("reservoir" OR "pipeline") AND ("biocorrosion" OR "microbiologically influenced corrosion" OR "microbial corrosion" OR ("MIC" AND "corrosion")) AND ("metagenomics" OR "16s rRNA") |
| PubMed | ("petroleum" OR "crude oil" OR "oilfield" OR "oil") AND ("reservoir" OR "pipeline") AND ("biocorrosion" OR "microbiologically influenced corrosion" OR "microbial corrosion" OR ("MIC" AND "corrosion")) AND ("metagenomics" OR "16s rRNA") |
| One Petro | ((petroleum) OR (crude oil) OR (oilfield) OR (oil)) AND ((reservoir OR pipeline)) AND ((biocorrosion) OR ("microbiologically influenced corrosion") OR ("microbial corrosion") OR (MIC AND corrosion)) AND ((metagenomics) OR ("16s rRNA")) |

**Supplementary material S1**: Table showing the search formats used in different databases.
